# A Comparative Study of Health Efficacy Indicators in Subjects with T2DM Applying Power Cycling to 12 Weeks of Low-Volume High-Intensity Interval Training and Moderate-Intensity Continuous Training

**DOI:** 10.1155/2022/9273830

**Published:** 2022-01-13

**Authors:** Jun Li, Wei Cheng, Haifeng Ma

**Affiliations:** ^1^School of Physical Education and Sport Training, Shanghai University of Sport, Shanghai 200438, China; ^2^Department of Endocrinology, Yangpu Hospital, School of Medicine, Tongji University, Shanghai, 200090, China

## Abstract

This study is aimed at comparing the effects of different exercise intensities, namely, high-intensity interval training (HIIT) and moderate-intensity continuous training (MICT), on body composition, heart and lung fitness, and blood glucose, and blood pressure indices in patients with type 2 diabetes mellitus (T2DM), using power cycling. A total of 96 T2DM volunteers who met the inclusion criteria were recruited from a hospital in Yangpu, Shanghai. Based on the blood index data of their medical examination results which comprised blood pressure, fasting blood glucose, hemoglobin A1c (HbA1c), and insulin, 37 volunteers were included in the study. Exercise prescription was determined based on T2DM exercise guidelines combined with medical diagnosis and exercise test results, and the patients were randomly assigned to three groups: HIIT group, MICT group, and control (CON) group. HIIT involved one-minute power cycling (80%–95% maximal oxygen uptake (VO2max)), one-minute passive or active rest (25%–30% VO2max), and two-minute rounds of eight groups. MICT required the use of a power bike for 30 minutes of continuous training (50%–70% VO2max) five times a week. The CON group was introduced to relevant medicine, exercise, and nutrition knowledge. The exercise interventions were completed under the supervision of an exercise instructor and hospital doctors. The same indicators were measured after 12 weeks of intervention, and the results of the two tests within and between groups were analyzed for comparison. The weight index of the MICT intervention showed statistically significant within-group differences (difference = 3.52, 95% CI = 2.11–4.92, *p* = 0.001 < 0.01); group differences for the MICT and CON groups were also statistically significant (difference = 3.52 ± 2.09, Cd1 = −0.39 ± 1.25, *p* = 0.004 < 0.01). Body mass index (BMI) analysis revealed that the overall means of BMI indicators were not statistically different between groups (*F* = 0.369, *p* = 0.694 > 0.05) and the before and after values of the MICT and CON (difference = −1.30 ± 0.79, Cd1 = −0.18 ± 0.45, *p* = 0.001 < 0.01). No statistically significant difference was observed in the overall mean VO_2_max index between the groups after the 12-week intervention (*F* = 2.51, *p* = 0.100 > 0.05). A statistically significant difference was found in the overall means of the data between the two groups (difference = 0.32, 95% CI = 0.23–0.40, *p* = 0.001 < 0.01). Analysis of fasting blood glucose (FBG) indicators revealed statistically significant differences between the MICT and control groups (*p* = 0.028 < 0.05). Analysis of HbA1c and fasting insulin (FI) indicators revealed no statistically significant difference in the overall HbA1c index after the 12-week exercise intervention (*F* = 0.523, *p* = 0.598 > 0.05), and the overall difference before and after the experiment between the groups was statistically significant (*F* = 6.13, *p* = 0.006 < 0.01). No statistically significant difference was found in the FI index overall after the 12-week exercise intervention (*F* = 2.50, *p* = 0.1 > 0.05). Analysis of systolic blood pressure (SBP) revealed statistically significant difference before and after the HIIT and CON interventions (Hd7 = −1.10 ± 1.79, Cd7 = 1.2 ± 1.31, *p* = 0.018 < 0.05) and statistically significant difference before and after the MICT and CON interventions (Md7 = −0.99 ± 0.91, Cd7 = 1.40 ± 1.78, *p* = 0.02 < 0.05). The diastolic blood pressure (DBP) revealed no statistically significant within-group differences before and after. Exercise interventions applying both low-volume HIIT and MICT, with both intensity exercises designed for power cycling, improved health-related indicators in the participants; low-volume HIIT had more time advantage. The current experiment compared HIIT with MICT in a safe manner: 50% of the exercise time produced similar benefits and advantages in the two indicators of VO_2_max and FI. However, MICT was superior to HIIT in the two indicators of body weight (weight) and BMI. The effect of power cycling on FI has the advantages of both aerobic and resistance exercise, which may optimize the type, intensity, and time of exercise prescription according to the individual or the type of exercise program. Our results provide a reference for the personalization of exercise prescription for patients with T2DM.

## 1. Introduction

Diabetes is a major public health problem worldwide [1], with type 2 diabetes mellitus (T2DM) accounting for more than 90% of cases. Diabetes is accompanied by a range of risk factors, such as cardiovascular diseases, hypertension, and dyslipidemia. Even with supplemental medications, the economic and social problems associated with T2DM treatment are becoming increasingly serious. With the multifaceted nature of T2DM etiology, with modifiable factors such as being overweight/obese, physical inactivity, and sedentary lifestyle, reducing the burden of disease requires effective and accessible lifestyle interventions [2, 3].

Exercise interventions are an important tool in the prevention and management of T2DM [4, 5] and can improve a wide range of cardiovascular and metabolic outcomes [6–8]. However, the optimal exercise prescription to maintain or improve the health status of the T2DM population remains uncertain. Exercise guidelines generally recommend at least 150 minutes of continuous moderate intensity (40%–60% of maximal oxygen uptake (VO2max)) or 75 minutes of higher-intensity (60%–85% VO2max) exercise per week [9, 10], but existing strategies face significant challenges, from lack of adherence to limited motivation and time to follow these guidelines [11]. The primary goal of exercise interventions in T2DM is to improve glycemia and insulin levels, but given the presence of comorbidities and the variety of causative factors in a larger T2DM population, improving body composition, aerobic fitness, and blood pressure and lipid levels are also important goals [12]. The increase in the prevalence of T2DM calls for more effective and targeted exercise prescriptions, and the type and intensity of exercise training should be tailored to the patient. Although the range of exercise guidelines for the T2DM population is currently expanding, specific evidence on the most recommended frequency, intensity, duration, and type of exercise for different conditions is lacking [13]. Therefore, understanding the different variables associated with the beneficial effects of exercise in the T2DM population is of practical interest [14, 15]. High-intensity interval training (HIIT) appears to be a viable and effective alternative exercise regimen to traditional moderate-to-high-intensity continuous training. It consists of alternating repetitions of short periods of high-intensity exercise interspersed with less active or passive recovery periods. HIIT has a moderating effect on clinical measures of the T2DM population and has also demonstrated effectiveness on glycemia, insulin, body composition, blood pressure, and aerobic capacity levels [16]. HIIT and MICT have different characteristics and effects as two training modalities, but there is not enough published data to conclude which is more effective [17]. Recently, published meta-analyses of the effects of HIIT practice have only demonstrated that HIIT is superior to MICT in improving cardiorespiratory fitness in patients with T2DM [18]. Body composition, blood glucose, and blood pressure were not found to differ between the two exercise modalities. Moreover, the high risk of study bias and low quality of evidence require the use of more randomized controlled trials [19].

As HIIT has several contraindications and risks in the T2DM population [20], it should only be recommended if the benefit or motivation is at least similar to that of MICT [10, 20, 21], and the potential benefits of HIIT should be compared with conventional MICT to ensure the best possible health benefits. Thus, people with T2DM may choose a more optimal intensity exercise prescription based on their own motivation or on the goal of improving different health indicators. Gibala et al. built on the original study by examining the impact of a lighter intensity exercise, a more realistic and feasible HIIT protocol [22, 23], proposed a low-volume HIIT protocol consisting of ten 60-second work rounds at 90% of maximum heart rate with 60-second recovery intervals, which could provide a more suitable duration and a better level of motivation [24–26]. Many effective HIIT protocols involved high-intensity uphill treadmill walking or running, which may be difficult for people with T2DM who are at a higher risk of falls and have limited lower extremity mobility. Thus, it is difficult to compare studies on the modalities, timing, and environmental conditions of exercise prescriptions needed to optimize efficacy [27].

Power cycling is desirable and commonly ideal in settings such as physical fitness and rehabilitation centers. This exercise mobilizes large muscle groups without weight bearing and foot-to-ground friction during running. Especially for the T2DM population, it is necessary to determine the HIIT and MICT protocols before applying power cycling for safety and effectiveness purposes. The present study is aimed at implementing a low-volume HIIT and MICT exercise intervention using power bikes as exercise equipment to provide evidence for HIIT and MICT exercise management and optimal exercise prescription in the T2DM population. Specifically, it aimed to (1) apply power cycling to the T2DM population for HIIT and MICT experimental data collection to test for good tolerance and (2) compare the different effects of HIIT and MICT exercise interventions on body composition, aerobic fitness, blood glucose, insulin, and other important indicators of health in a T2DM population.

## 2. Materials and Methods

### 2.1. Participant Inclusion and Exclusion

**Figure 1:**
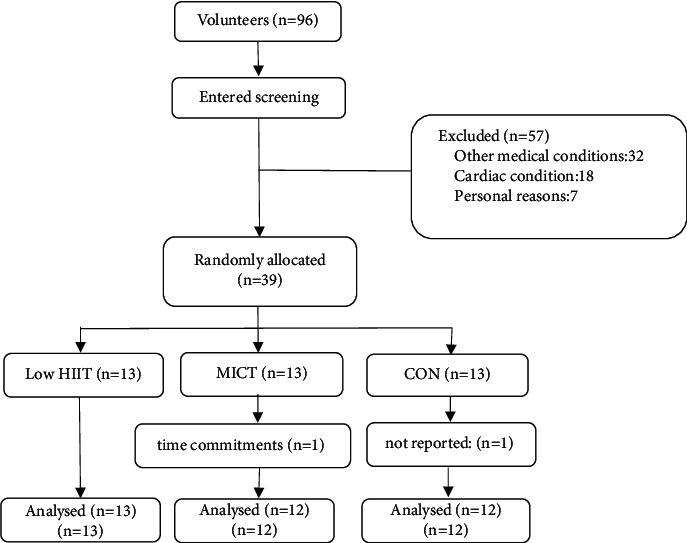
Flow chart of volunteer recruitment for the experiment.

Thirty-seven male volunteers were recruited from the diabetes clinic of a hospital in Yangpu District, Shanghai, according to the succeeding criteria. The following were screen for the physical examination: diagnosis of T2DM for at least one year, meeting the WHO diagnostic criteria for diabetes [24], no major macroscopic or microscopic vascular complications of diabetes, aged 32 to 47 years, and with a body mass index (BMI) of <35 kg/m^2^. Participants also must have no physical limitations to the exercise intervention to be performed, no limitations in gait or balance, and no major health problems. For the behavioral habits, the following were checked: no smoking in the past six months, no participation in a supervised exercise program, and maintenance of a diet for at least six months. All participants were asked to complete the Physical Activity Questionnaire, which includes eating behavior. The recruitment also included exercise testing. Prior to participation in the trial, all participants underwent a detailed medical assessment to screen for relative or absolute contraindications to high-intensity exercise, including the use of the exercise plate test (Bruce protocol) to confirm the absence of potential cardiac contraindications. Meanwhile, the exclusion criteria were as follows: participants receiving exogenous insulin therapy; smokers; those with unstable weight (5 kg/6 months); those with a condition that precluded physical activity, such as evidence of acute disease or renal, hepatic, or cardiovascular disease; failure to perform all experimental conditions; failure to complete changes in medications prescribed for the experiment period; changes in dietary patterns; and participation in other supervised exercises (see Figure 1).

The experimental procedures and potential risks were explained to the participants prior to the study. Written informed consent was obtained from all the participants. They received good treatment at baseline, and their medications were not changed during the study. The study protocol was approved by the local hospital ethics committee (Ethics Committee Protocol Number: LL-KY-009).

### 2.2. Study Methodology

#### 2.2.1. Experimental Design and Preexercise Adaptation

For participant allocation during the 12-week parallel randomized controlled clinical trial, after baseline assessment, researchers external to the project used a computer-generated random number list with a 1 : 1 : 1 allocation ratio. The participants were given opaque sealed envelopes and were randomized into three groups: HIIT (*n* = 13), MICT (*n* = 12), and CON (*n* = 12). Both the HIIT and MICT groups used the Swedish Monark power bike as a device for the exercise intervention, whereas the CON group received standard counseling on conventional T2DM exercise guidelines and did not perform organized exercise. For ethical reasons, all participants were provided with standard counseling on topics such as nutrition or exercise to improve trial adherence. Throughout the study period, participants also received information on maintaining activities of daily living (daily diet habitual physical activity and medication). Counseling covered overcoming barriers to exercise, enhancing self-regulation, self-efficacy, planning, and increasing awareness of the physical and mental benefits of exercise, considering that fluctuating blood glucose levels may be more harmful than stable high blood glucose levels. The HIIT, MICT, and CON groups were instructed in the 14-day real-time monitoring of blood glucose dynamics with a blood glucose monitor to avoid excessive fluctuations in blood glucose levels. They were given a detailed explanation of the heart rate scale, scores, and meaning of the ratings of perceived exercise (RPE) scale before the intervention. The patients were also allowed to rate themselves during the preexperiment to familiarize themselves with the form. They were introduced to their assigned exercise modality, namely, HIIT or MICT, to help them maintain consistent exercise adherence. Two weeks prior to the start of the study, the participants were organized to visit the laboratory and try the power bikes to help them acclimatize. The HIIT intensity was not standardized but based on the individual cardiorespiratory adaptations of the exercisers, and the experimental procedure was explained in detail to all participants. In the days leading up to the experiment, the participants were asked to maintain a normal diet and avoid engaging in extrasport exercise or strenuous physical activity. Participants in both exercise groups received 15 minutes of behavioral coaching three times a week (45 minutes in total) with the aim of preparing them for the transition to the exercise prescriptions by power cycling. The load used to achieve the different intensities of exercise tested in the study (HIIT and MICT) was progressively increased via one-on-one coaching.

#### 2.2.2. HIIT and MICT Exercise Protocol

**Figure 2:**
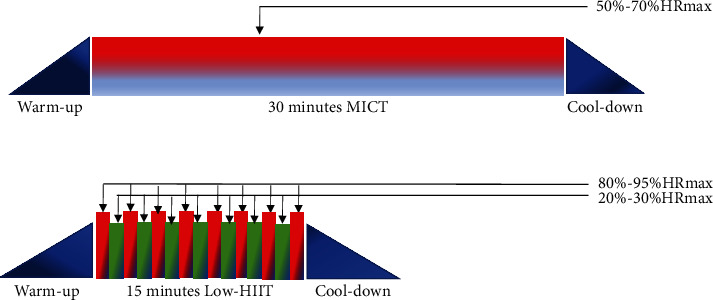
Schematic diagram of the two exercise schemes.

Both groups exercised for five times per week, supervised by an exercise instructor, and monitored using a heart rate band (polarT-31, USA). The maximum oxygen uptake of patients with T2DM was tested using the Astrand test method with the Swedish Monark power bike LC7, and their heart rate was monitored using a polar meter. A self-fatigue scale was placed directly in front of the power bike. The exercise intervention protocol was standardized according to extant guidelines [28] and consisted of 30 minutes per session for the MICT, except for preparatory and finishing activities, and 15 minutes per session for the HIIT group. This is consistent with the recommendations of at least 150 minutes of moderate-intensity exercise or 75 minutes of vigorous exercise per week for adults. All participants completed a five-minute warm-up and five-minute finishing activities at similar intensities during each supervised exercise session [29]. In summary, the time (minutes) allocated to each exercise session was as follows: a five-minute warm-up (three minutes off the bike, two minutes on the bike), moderate-intensity exercise (gradually adjusting the power bike load to a heart rate maximal oxygen uptake in the 50%–70% and 80%–95% range for MCT and HIIT, respectively) for 30 minutes for MICT and 15 minutes for HIIT (including eight minutes of intensity in the 80%–95% range and seven minutes of intervals for active recovery at approximately 25% intensity [30]), and five minutes to complete the relaxation and finishing process (see Figure 2).

#### 2.2.3. Supervision and Exercise Intervention Process

The participants were contacted to determine specific times for each exercise intervention, and the exercise intervention protocol was generally performed under the supervision of both an exercise instructor and a physician. The intervention had five scheduled exercise workouts per week with no more than two days of intervals between sessions, for a total of 60 scheduled workouts over the 12-week study. The HIIT and MICT protocols were maintained in the intensity range of one-two sessions over 12 weeks of training on an individual basis. Heart rate provided a basis for progression throughout the exercise intervention, with an increasing number of repetitions and duration of each repetition. Heart rate was continuously monitored during the supervised exercise intervention, and participants' heart rates were recorded using a downloadable Polar heart rate monitor (Polarft7, Finland) to ensure training at the intended intensity. During each session, the heart rate was recorded using a cycle tester. RPE was recorded using a subjective exertion rating scale (RPE 6-20) at the end of each week [12]. When a rapid rise in heart rate occurred during exercise, the researcher made inquiries and stopped the experiment if the participant reported discomfort. If chest tightness, heart pain, and head pain were reported, the experiment was stopped immediately. Participants were asked to engage in simple housework, walking, and other daily physical activities in addition to the HIIT and MICT exercise protocols during the exercise intervention.

#### 2.2.4. Tests of Basic Physical Characteristics, Blood Biochemical Indicators

All participants were tested for height, weight, blood pressure, VO2max, fasting blood glucose (FBG), glycated hemoglobin (HbA1c), fasting insulin (FI), and other blood biochemical indices at the experimental hospital before the exercise intervention was conducted. The participants' BMI was calculated, and the VO2max assessment test was performed using the Astrand test method available on the Swedish Monark power bicycle LC7. For glucose, the glucose oxidase method was used. For HbA1c, an affinity chromatography microcolumn assay was used. An enzyme-linked immunoassay using an automated biochemical analyzer was done to assess insulin level. The test indices were repeated after the 12-week experiment.

#### 2.2.5. Statistical Analysis

Data analysis was performed using IBM SPSS Statistics for Windows, Version 22.0, and all data were expressed as mean ± standard deviation. To compare the data before and after the intervention in each group, normality test was performed. Paired sample *t*-test was used for results following normal distribution, and Wilcoxon test for two associated samples was used for those that do not. For comparison between the HIIT, MICT, and CON groups after the intervention, data following the normal curve were subjected to single-factor ANOVA. The Bonferroni post hoc test was used for two-way comparisons. Those that did not follow a normal distribution were subjected to the Kruskal–Wallis *H*-test. All pairwise comparisons of Kruskal–Wallis and one-way ANOVA multiple comparisons were used for two-by-two comparisons. When ANOVA results were not statistically significant, one-way ANOVAs or rank-sum tests were used for before and after differences for each group. In all tests, statistical significance was set at *p* < 0.05.

## 3. Results

### 3.1. Participants' General Conditions

Table 1 shows the characteristics of the participants in the three groups. The participants' baseline conditions were not statistically different and were comparable in terms of basic information, body morphology, cardiopulmonary function, and glucose, insulin, and lipid metabolism levels (Table 2). No adverse events were observed or reported by participants throughout the course of the exercise training protocol.

### 3.2. Comparison of Weight, BMI, and VO_2_max Indicators between Groups after Exercise Intervention

The weight index values before and after MICT intervention were 73.12 ± 7.83 kg and 69.60 ± 5.91 kg, respectively. Statistical differences was observed within groups (difference = 3.52, 95% CI: 2.11–4.92, *p* = 0.001 < 0.01). The mean weight index did not differ significantly between the groups (*F* = 0.953, *p* = 0.398 > 0.05). The difference weight before and after the experiment was statistically different overall among the three groups (*F* = 12.90, *p* = 0.002 < 0.01), with differences observed before and after the experiment between HIIT and MICT (Hd = −0.51 ± 1.04, Md = 3.52 ± 2.09, *p* = 0.011 < 0.05), before and after the experiment between HIIT and CON (Hd = −0.72 ± 0.35, Cd = −0.39 ± 1.25, *p* = 1.000 > 0.05), and before and after the MICT and CON experiments (Md = 3.52 ± 2.09, Cd = −0.39 ± 1.25, *p* = 0.004 < 0.01).

**Figure 3:**
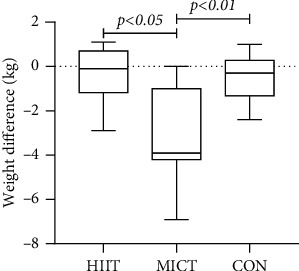
Comparison of weight difference between groups after the intervention.

**Figure 4:**
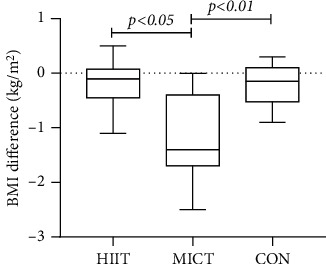
Comparison of BMI differences in each group after the intervention.

BMI index analysis revealed a range of 26.75 ± 4.20 kg/m^2^ before the MICT exercise intervention and a range of 25.45 ± 3.51 kg/m^2^ 12 weeks after the exercise intervention. The overall means of the data in the HIIT and MICT groups were statistically different (difference = 1.3, 95% CI: 0.77–1.83, *p* = 0.001 < 0.01). The overall means of BMI indicators were not statistically different between the groups (*F* = 0.369, *p* = 0.694 > 0.05). The overall pre- and postexperimental difference d2 of the groups was statistically different (*F* = 13.02, *p* = 0.001 < 0.01) between HIIT and MICT, (Hd = −0.21 ± 0.37, Md = −1.30 ± 0.79, *p* = 0.001 < 0.05), between HIIT and CON (Hd = −0.21 ± 0.37, Cd = −0.18 ± 0.45, *p* = 1.000 > 0.05), and between MICT and CON (Md = −1.30 ± 0.79, Cd = −0.18 ± 0.45, *p* = 0.001 < 0.01) (see Figures 3 and 4) (note: HIIT: high-intensity interval training; MICT: moderate-intensity continuous training; CON: control).

**Figure 5:**
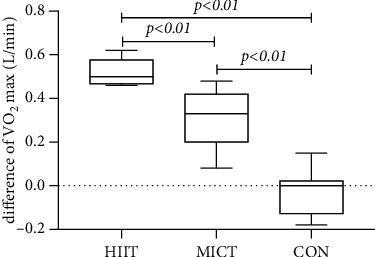
Comparison of difference of VO_2_max in each group after intervention.

The within-group MICT exercise VO_2_max indicators before and after the intervention in each group were 3.46 ± 0.38 L/min and 3.77 ± 0.45 L/min, respectively, with a statistically significant difference in the overall means of the data between the two data sets (difference = 0.32, 95% CI: 0.23–0.40, *p* = 0.001 < 0.01). The HIIT exercise VO_2_max index was 3.39 ± 0.44 L/min before and 3.92 ± 0.43 L/min after the intervention, with a statistically significant difference in overall means between the two data sets (difference = 0.53, 95% CI: 0.48–0.57, *p* = 0.001 < 0.01). There was no statistically significant difference in the overall mean VO_2_max index between the groups after the 12-week intervention (*F* = 2.51, *p* = 0.100 > 0.05). The pre- and postexperimental differences were statistically different overall (*F* = 75, *p* = 0.001 < 0.01), with pre- and postexperimental differences between HIIT and MICT (Hd = 0.52 ± 0.06, Md = 0.31 ± 0.13, *p* = 0.001 < 0.01), between HIIT and CON (Hd = 0.52 ± 0.06, Cd = −0.03 ± 0.10, *p* = 0.001 < 0.01), and before and after the MICT and CON (Md = 0.31 ± 0.13, Cd = −0.03 ± 0.10, *p* = 0.001 < 0.01) (see Figure 5) (note: HIIT: high-intensity interval training; MICT: moderate-intensity continuous training; CON: control).

### 3.3. Comparison of Blood Glucose and Insulin Indices between Groups after Exercise Intervention

The within-group FBG indicators were 7.60 ± 0.52 mmol/L before and 6.83 ± 0.44 mmol/L after the exercise intervention for MICT, with a statistically significant difference in the overall means of the data between the two data sets (difference = 0.77, 95% CI: 0.49–1.05, *p* = 0.001 < 0.01). The HIIT FBG index was 7.80 ± 0.50 mmol/L before and 6.93 ± 0.33 mmol/L after the exercise intervention, with a statistically significant difference in overall means between the two data sets (difference = 0.87, 95% CI: 0.66–1.07, *p* = 0.001 < 0.01). The overall means of the FBG index were statistically different between groups after the 12-week intervention (*F* = 4.399, *p* = 0.022 < 0.05), with the MICT showing statistically different results from the control group (*p* = 0.028 < 0.05).

**Figure 6:**
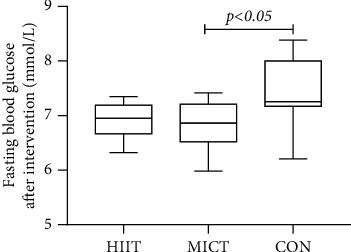
Fasting blood glucose values of each group after intervention.

**Figure 7:**
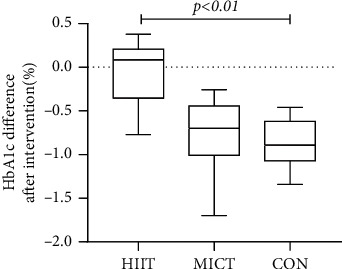
Difference of HbA1c in each group after intervention.

Analysis of HbA1c indicators revealed that the overall mean was statistically different between the HIIT and MICT groups (difference = 0.14, 95% CI: 0.04–0.23, *p* = 0.009 < 0.01) after the intervention. For the MICT group, the indicator was 7.02 ± 0.44 mmol/L before the intervention and 6.88 ± 0.40 mmol/L after. The HIIT HbA1c index was 7.18 ± 0.50 mmol/L before and 6.79 ± 0.41 mmol/L after, with a statistically significant difference in overall means between the two data sets (difference = 0.21, 95% CI: 0.07–0.34, *p* = 0.009 < 0.01). No statistically significant difference was found in the overall HbA1c index after the 12-week exercise intervention (*F* = 0.523, *p* = 0.598 > 0.05). The overall difference in before and after the experiment was statistically different between HIIT and MICT (*F* = 6.13, *p* = 0.006 < 0.01). The differences before and after the experiment were as follows: between HIIT and MICT (Hd = −0.20 ± 0.19, Md = −0.14 ± 0.14, *p* = 0.972), between HIIT and CON (Hd = −0.20 ± 0.19, Cd = 0.03 ± 0.12, *p* = 0.006 < 0.01), and between MICT and CON (Md = −0.14 ± 0.14, Cd = 0.03 ± 0.12, *p* = 0.06 > 0.05) (see Figures 6 and 7) (note: HIIT: high-intensity interval training; MICT: moderate-intensity continuous training; CON: control).

**Figure 8:**
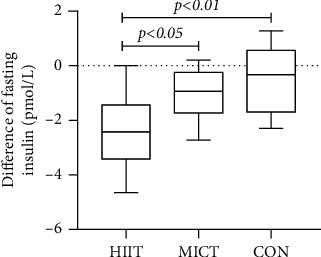
Difference in fasting insulin levels in each group after intervention.

The FI indicators were 26.35 ± 1.43 pmol/L before and 25.35 ± 1.49 pmol/L after the MICT exercise intervention, with a statistically significant difference in the overall means of the data between the two data sets (difference = 0.99, 95% CI: 0.38–1.60, *p* = 0.005 < 0.01). The FI index was 27.04 ± 1.06 pmol/L before and 24.65 ± 1.38 pmol/L after the HIIT exercise intervention, with a statistically significant difference between the overall means of the two data sets (difference = 0.51, 95% CI: -0.39–1.41, *p* = 0.001 < 0.01). There was no statistically significant difference noted in the FI index overall after the 12-week exercise intervention (*F* = 2.50, *p* = 0.1 > 0.05). The pre- and postexperimental differences were statistically different overall between the groups (*F* = 6.37, *p* = 0.005 < 0.01), between HIIT and MICT (Hd = −2.39 ± 1.47, Md = −0.99 ± 0.91, *p* = 0.043 < 0.05), between HIIT and CON (Hd = −2.39 ± 1.67, Cd = −0.51 ± 1.26, *p* = 0.006 < 0.01), and between MICT and CON (Md = −0.99 ± 0.91, Cd = −0.51 ± 1.26, *p* = 1.000 > 0.05) (see Figure 8) (note: HIIT: high-intensity interval training; MICT: moderate-intensity continuous training; CON: control).

### 3.4. Comparison of Blood Pressure Indicators between Groups after Exercise Intervention

**Figure 9:**
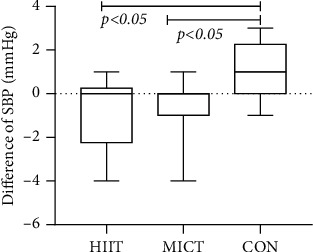
Blood pressure differences of each group after intervention.

Analysis of systolic blood pressure indicators revealed ranges of 134.2 ± 8.4 mmHg before and 135.4 ± 8.9 mmHg after the CON exercise intervention, with a statistical difference in the overall mean of the data between the two (difference = 1.2, 95% CI: 0.26–2.14, *p* = 0.018 < 0.05). Twelve weeks of systolic blood pressure (SBP) showed no overall statistical difference after the exercise intervention (*F* = 1.44, *p* = 0.25 > 0.05). The overall pre- and postexperimental difference was statistically different between the three groups (*F* = 5.766, *p* = 0.008 < 0.01): between HIIT and MICT (Hd7 = −0.8 ± 1.69, Md7 = −0.73 ± 1.49, *p* = 1.000 > 0.05), between HIIT and CON (Hd7 = −1.10 ± 1.79, Cd7 = 1.2 ± 1.31, *p* = 0.018 < 0.05), and between MICT and CON (Md7 = −0.99 ± 0.91, Cd7 = 1.40 ± 1.78, *p* = 0.02 < 0.05). Analysis of diastolic blood pressure indices revealed no statistically significant differences within the group before and after, in DBP indexes after 12 weeks of exercise intervention (*F* = 1.325, *p* = 0.282 > 0.05), and overall in between the three experimental groups before and after exercise (*F* = 1423, *p* = 0.258 > 0.05) (see Figure 9) (note: HIIT: high-intensity interval training; MICT: moderate-intensity continuous training; CON: control).

## 4. Analysis and Discussion

HIIT has been proven effective or even has superior effects in the T2DM population in numerous studies. However, different exercise types, programs, intensities, and durations can have different effects, and the optimal exercise prescription still needs to be validated for different conditions. This study was designed to apply power cycling to compare the effects of low-volume HIIT and MICT aerobic exercise interventions on health-related indicators in a T2DM population. Participants were divided into HIIT, MICT, and CON groups by preexercise testing and were then randomly assigned to risk factors associated with T2DM and comorbidities, namely, basic body composition, cardiorespiratory fitness, glucose level, insulin level, and blood pressure. To investigate whether HIIT can be an effective alternative or an optimized protocol for MICT in the T2DM population with respect to different indicators, a lighter and more practical and feasible HIIT protocol was designed. This randomized controlled trial showed that under effective supervision, the application of low-volume HIIT or MICT on power bikes is safe, feasible, and well-tolerated in the T2DM population. Moreover, 12-week HIIT and MICT programs are not only effective but also ideal for targeting different health indicator.

### 4.1. Safe, Feasible, and Well Tolerated Exercise

The exercise protocol was performed under professional supervision. The application of power bikes considered not only the mobilization of large muscle groups and the absence of weight bearing on the legs and running friction on the ground. The HIIT intervention was also designed to be lower in volume and practically feasible. The total exercise time complied with the exercise guidelines of no less than 75 minutes per week at a high-intensity standard relative to the MICT. The training time was reduced by 50%. The complete experimental process included two late exclusions for personal objective reasons. The supervision records showed no adverse reactions or negative emotions during the experiment, and the interventions were well tolerated.

### 4.2. Response and Comparison of HIIT and MICT on Health-Related Indicators in the T2DM Population

Weight and BMI indicators are associated with an increased risk of cardiovascular complications in people with T2DM [31–33]. Weight loss mitigates the risk associated with diabetes [28, 34], and substantial weight loss can achieve long-term remission of diabetes [35, 36]. BMI is an indicator of general obesity and has been widely used to determine obesity and overweight in the population [37, 38]. Moreover, BMI has been linked to the time of diabetes diagnosis and the risk of death [39, 40]. Statistical differences in weight and BMI were found before and after the intervention in the MICT group, whereas only BMI was statistically different in the HIIT group. Weight loss from exercise training is mainly attributed to the energy expenditure accumulated during actual exercise [41]. The HIIT duration may have been too short in this study, resulting in limited energy expenditure. The effect of MICT exercise on weight loss in the participants was highlighted in the recorded weight loss, which also correlated with T2DM exercise guidelines (at least 150 minutes of moderate sustained and 75 minutes of high-intensity exercise are recommended). However, at least 200 minutes of moderate sustained training per week is recommended by the exercise guidelines for weight loss. Indeed, studies have shown that 150 minutes of moderate-intensity exercise per week is insufficient for weight loss [42]. Obesity is a key contributor to type 2 diabetes and may be the first factor to consider when encountering exercise prescription settings for such populations. However, this experiment was intended to compare relevant health indicators, not weight loss. These results could not adequately reflect the differential effects of body fat changes. First, although the cumulative weekly MICT exercise time was less than the minimum time standard recommended by the 200-minute weight loss exercise guidelines, the cumulative physical activity of preparation and finishing activities and daily living to achieve MICT may exceed 200 minutes. Moreover, the participants were encouraged to maintain good dietary and lifestyle habits during the experimental period. Second, participants in all groups were generally overweight, and exercise weight loss had a greater intervention effect on individuals with greater initial adiposity [43]. Considering factors such as the duration of obesity in the T2DM population and the fact that HIIT was conducted for half the time compared with the MICT group, the HIIT intervention could have shown similar effects on BMI indicators and a greater tendency to reduce BMI over a short period of time. Nonetheless, the insignificant change in HIIT weight was not unexpected, as one study found increased energy expenditure during the recovery phase of HIIT [44] and postexercise. Elevated plasma catecholamine levels in HIIT-driven lipolysis [45] are signs that HIIT affects appetite-regulating hormones, and that weight loss effects include a reduction in appetite after exercise in individuals. Similarly, studies [46] have stated that for patients with common metabolic disorders, such as T2DM, there is insufficient evidence that HIIT reduces weight compared with MICT, and that the role of HIIT in weight loss should not be exaggerated. The statistical differences in the pre- and postexperiment weight and BMI indicators between groups suggested that the current exercise duration and minimum energy expenditure standards for the HIIT training modality may be insufficient to have a significant weight loss effect. Future studies should clarify whether HIIT can provide an effective means of weight loss in patients with T2DM and elucidate the duration and intensity of HIIT training to be conducted.

VO2max is an important indicator for assessing cardiorespiratory endurance [19].

Low aerobic exercise capacity appears to be the strongest predictor of mortality among all known risk factors. Decreased cardiorespiratory fitness is common in patients with T2DM [47] and is strongly associated with mortality [48, 49]. Regular aerobic exercise, including the major muscle groups of the legs, arms, and trunk, is recommended to improve cardiorespiratory endurance capacity [50]. Maintaining or improving cardiorespiratory endurance in patients with T2DM is of great importance. In the present study, VO2max increased by 0.53 L/min in the HIIT group and by 0.32 L/min in the MICT group after the 12-week intervention. These results are clinically important because an increase of 0.35 L/min is associated with a 15% reduction in all-cause mortality and a 19% reduction in cardiovascular disease mortality [51]. In addition, higher aerobic capacity is associated with a higher quality of life in the T2DM population [52]. According to a recent meta-analysis comparing HIIT and MICT in the T2DM population, HIIT was significantly better than MICT relative to VO2max metrics. Nonetheless, evidence shows that in patients with cardiometabolic diseases, such as T2DM, vigorous or high-intensity exercise may lead to an increased risk of adverse effects (e.g., atrial tachycardia and myocardial infarction), at least temporarily [53]. The present study showed that both HIIT and MICT resulted in greatly improved VO2max in patients with T2DM. The effect of HIIT on VO2max was greater in the HIIT group than in the CON group, but the HIIT group did not show a significantly better increase in VO2max than the MICT group similar to other studies. In conventional studies, the maximum oxygen uptake of HIIT was significantly better than that of MICT. This inconsistency may be due to the following reasons. First, the power cycling participants may have had difficulty fully opening their chest with their upper body low on the handrail, creating a limitation on breathing and resulting in a lower expected increase in VO2max compared with running exercises. However, performing high-intensity intervals on a power bike reduced the risk of foot and ground friction and muscle or joint injury that tended to occur while running at high speed on the ground. Second, differences in type, program, and length of HIIT exercise could explain the uneven VO2max improvement and would require further in-depth study. Therefore, the results of this experiment suggest that the interventions were effective in improving VO2max in the T2DM population. The statistical difference between the pre- and postintervention VO2max indices indicates an advantage of HIIT, but it is not significant compared with other studies.

Both MICT and HIIT lowered fasting blood glucose in patients with T2DM and similar results were observed in a previous study. However, MICT required 45% more time than the HIIT protocol, and results of the current study emphasized the efficiency of HIIT in producing comparable effects on fasting blood glucose to MICT [54] in a more time-efficient manner. HIIT exercise on a cycle ergometer has been found to have no effect in terms of altering fasting blood glucose levels in subjects with T2DM [16]. However, most studies have shown that HIIT is an effective strategy for reducing fasting blood glucose concentrations in patients with T2DM. This study was not specifically designed to examine the effects of HIIT and MICT on glycemic control but rather to compare the inconsistent glycemic outcomes of MICT and HIIT on exercise interventions in the T2DM population. The main findings of this study were that (1) there was a statistical difference in fasting blood glucose for both MICT and HIIT before and after the intervention, with no statistical difference in fasting blood glucose between the two exercise intensities in a cross-sectional comparison after the intervention, and (2) there was a statistical difference with the control for MICT. Therefore, to achieve glycemic control in people with T2DM, easy-to-implement MICT can replace the more physically demanding HIIT.

HbA1c is not only the most widely used glycemic indicator but is also an important risk factor for cardiovascular disease in patients with T2DM. A 1% reduction in glycosylated hemoglobin levels is associated with a 37% reduction in the risk of microvascular complications and a 21% reduction in the risk of diabetes-related death [55, 56]. The lifespan of red blood cells is approximately four months, but many exercise training studies have been conducted for a shorter duration. HbA1c is a blood marker that quantifies the three-month average blood glucose concentration. Intuitively, an exercise program may take longer than 12 weeks to demonstrate an effect on HbA1c, yet many studies have a duration of 12 weeks or shorter. One study concluded that HbA1c decreases with each additional week of exercise compared with controls, and any reduction in HbA1c levels may reduce the risk of macrovascular and microvascular complications in patients with T2DM. Although this effect is small, it emphasizes the importance of sustained exercise interventions to improve health [16]. The present study found a statistical difference between the HIIT and CON groups only after the exercise intervention, probably because of (1) the duration of the exercise intervention and preparation time was approximately 13 weeks, and HIIT had an effect on HbA1c during the intervention period, and (2) the intensity difference. This experiment considered HIIT compared with MICT and found HIIT superior to MICT for HbA1c with consideration for intervention duration and effect.

Elevated FI has been suggested as a possible independent predictor of T2DM development [57]. Researchers have applied HIIT and MICT interventions to participants with T2DM after two randomized controlled trials: one experiment was superior in FI concentration, and the other was similar to MICT, which the authors attributed to the fact that both exercises improved insulin signaling in the skeletal muscle rather than to the effect of insulin secretion from pancreatic *β*-cells [10, 58]. Another meta-analysis comparing the effects of two different resistance intensities on FI in people with T2DM showed that resistance exercise leads to a significant reduction in FI only at high intensities, and that low to moderate intensities had no effect. The increase in GLUT4 protein levels in the skeletal muscle after strength training may be responsible for the enhanced insulin action in patients with T2DM [59]. Resistance exercise may improve insulin levels by increasing GLUT4 protein expression and insulin signaling without increasing muscle mass [15]. Based on the fact that different types of exercise lead to a possible improvement in FI for skeletal muscle molecular mechanisms, HIIT resistance exercise may be a primary consideration for achieving FI reduction. The statistical difference between the pre- and postintervention FI in the three groups after 12 weeks in this study showed superior HIIT effects, possibly because power cycling requires exerting large muscle groups aerobically, with the improved leg muscle strength producing similar effects to resistance exercise.

Both HIIT and MICT modalities had similar effects on systolic and diastolic blood pressure in patients with prediabetes and T2DM. Elevated blood pressure is common in diabetic patients and is considered a strong risk factor for atherosclerotic cardiovascular disease, heart failure, and microvascular complications. The present experiment concluded that both exercise modalities had a blood pressure reduction effect. It is difficult to distinguish between specific advantages and disadvantages, but both can maintain blood pressure stability. Moreover, the control group showed some control over blood pressure increase.

## 5. Conclusions

Exercise interventions applying both low-volume HIIT and MICT designed for power cycling improved health-related indicators in subjects with T2DM. Notably, HIIT showed a temporal advantage. The current experiment compared HIIT with MICT. HIIT, which required 50% of the exercise time of MICT, produced similar benefits as MICT and advantages in the two indicators of VO2max and FI. However, MICT was superior to that of HIIT in terms of body weight and BMI. The effect of cycling on FI demonstrated the advantages of both aerobic and resistance exercise, which may optimize the type, intensity, and time of exercise prescription in the future according to the individual or the type of exercise program. These findings can provide a reference for the personalization of exercise prescriptions for patients with T2DM.

## Figures and Tables

**Table 1:** Participant characteristics.

	HIIT	MICT	CON
Age (years)	38 ± 6	39 ± 5	40 ± 7
Height (cm)	166.9 ± 6.25	165.8 ± 5.56	166.7 ± 6.86
Time since diagnosis (years)	1.95 ± 0.55	1.79 ± 0.52	1.84 ± 0.49
Sex (m/f)	13	12	12
BMI	27.38 ± 5.53	26.75 ± 4.20	26.45 ± 4.97
Diet only	10	8	9
Comorbidities	3	4	3
Medication			
Metformin	6	6	7
Sulfonylureas	3	2	3
DPP-4 inhibitors	3	2	2
Alpha-glucosidase inhibitor	1		
Smokers	13/5	12/5	12/4

HIIT: high-intensity interval training; MICT: moderate-intensity continuous training; CON: control.

**Table 2:** Changes in participants' characteristic body composition, VO2max peak, blood glucose, and blood pressure control.

Groups	Pre	Post	*d*	*t*	*p*
Body mass (kg)					
HIIT	75 ± 9.98	74.49 ± 9.15	−0.51 ± 1.04		
MICT	73.12 ± 7.83	69.60 ± 5.91^∗∗^	−3.52 ± 2.09§	5.56	0.001
CON	71.76 ± 9.72	71.37 ± 9.23	−0.39 ± 1.25		
*F*			12.90		
*p*			0.002		
BMI (kg/m^2^)					
HIIT	27.38 ± 5.53	27.17 ± 5.23	−0.21 ± 0.37		
MICT	26.75 ± 4.20	25.45 ± 3.51^∗∗^	−1.30 ± 0.79§	5.46	0.001
CON	26.45 ± 4.97	26.27 ± 4.88	−0.18 ± 0.45		
*F*			13.02		
*p*			0.001		
VO2max (L/min)					
HIIT	3.4 ± 0.4	3.9 ± 0.4^∗∗^	0.52 ± 0.06§§	-27.29	0.001
MICT	3.5 ± 0.4	3.7 ± 0.5^∗∗^	0.31 ± 0.13	-8.06	0.001
CON	3.5 ± 0.4	3.5 ± 0.5	−0.03 ± 0.10		
*F*			75.00		
*p*			0.001		
Fasting glucose (mmol/L)					
HIIT	7.80 ± 0.50	6.93 ± 0.33^∗∗^#		9.70	0.001
MICT	7.60 ± 0.52	6.83 ± 0.44^∗∗^		6.07	0.001
CON	7.47 ± 0.57	7.42 ± 0.62			
*F*		4.39			
*p*		0.022			
HbA1c (%)					
HIIT	7.18 ± 0.50	6.79 ± 0.41^∗∗^	−0.20 ± 0.19	3.32	0.009
MICT	7.02 ± 0.44	6.88 ± 0.40^∗∗^	−0.14 ± 0.14	3.26	0.009
CON	7.06 ± 0.38	7.09 ± 0.33	0.03 ± 0.12		
*F*			6.13		
*p*			0.006		
Fasting insulin (pmol/L)					
HIIT	27.04 ± 1.06	24.65 ± 1.38^∗∗^	−2.39 ± 1.47§	5.14	0.001
MICT	26.35 ± 1.43	25.35 ± 1.49^∗∗^	−0.99 ± 0.91	3.63	0.005
CON	26.88 ± 1.64	26.37 ± 2.21	−0.51 ± 1.26		
*F*			6.37		
*p*			0.005		
Systolic (mmHg)					
HIIT	140.2 ± 3.23	139.4 ± 2.88			
MICT	135.5 ± 7.23	134.7 ± 7.02			
CON	134.2 ± 8.44	135.4 ± 8.85^∗^		-2.88	0.018
*F*					
*p*					
Diastolic (mmHg)					
HIIT	75.40 ± 6.55	74.50 ± 5.19			
MICT	74.55 ± 6.92	74.45 ± 5.92			
CON	77.80 ± 6.29	78.10 ± 6.21			
*F*					
*p*					

Values are mean ± standard deviation; paired-sample *t*-test was used to compare data before and after the intervention in each group, ^∗^*p* < 0.05, ^∗∗^*p* < 0.01 for statistically significant differences; one-way ANOVA was used to compare postintervention differences between HIIT, MICT, and CON groups, and Bonferroni post hoc test was used for two-way comparisons. #*p* < 0.05, ##*p* < 0.01 for statistically significant differences between groups; §*p* < 0.05, §§*p* < 0.01 for statistically significant differences in HIIT vs. MICT differences. HIIT: high-intensity interval training; MICT: moderate-intensity continuous training; CON: control.

## Data Availability

The data used to support the findings of this study are available from the request.

## Abbreviations

T2DM: Type 2 diabetes mellitus
HIIT: High intensity interval training
MICT: Moderate intensity continuous training
CON: Control group
BW: Body weight
BMI: Body mass index
VO2max: Maximal oxygen uptake
FBG: Fasting blood glucose
HbA1c: Glycated hemoglobin type A1C
FI: Fasting insulin
SBP: Systolic blood pressure
DBP: Diastolic blood pressure
HRmax: Maximal heart rate.
